# Spontaneous cervicocephalic arterial dissection with headache and neck pain as the only symptom

**DOI:** 10.1007/s10194-012-0420-2

**Published:** 2012-02-17

**Authors:** Hajime Maruyama, Harumitsu Nagoya, Yuji Kato, Ichiro Deguchi, Takuya Fukuoka, Yasuko Ohe, Yohsuke Horiuchi, Tomohisa Dembo, Akira Uchino, Norio Tanahashi

**Affiliations:** 1 Department of Neurology and Cerebrovascular Medicine, Saitama Medical University International Medical Center, 1397-1 Yamane, Hidaka, Saitama 350-1298 Japan; 2 Department of Diagnostic Radiology, Saitama Medical University International Medical Center, Hidaka, Japan

**Keywords:** Spontaneous cervicocephalic arterial dissection, Headache, Neck pain

## Abstract

**Background and objective:**

Cervicocephalic arterial dissection can cause both ischemic stroke and hemorrhagic stroke. However, spontaneous cervicocephalic arterial dissection presenting only with headache and neck pain has rarely been reported. The clinical features of patients with spontaneous cervicocephalic arterial dissection presenting only with headache and neck pain were investigated.

**Methods:**

The subjects were seven patients with spontaneous cervicocephalic arterial dissection with headache and neck pain alone who were admitted to our hospital during the past 3 years. The clinical features of these patients were investigated. The diagnosis of arterial dissection was based on the criteria of the Strategies Against Stroke Study for Young Adults in Japan.

**Results:**

The age of the patients (3 males, 4 females) ranged from 35 to 79 (mean, 51.0 ± 16.2) years. Six patients had vertebral artery dissection, one had internal carotid artery dissection, and one had an association of vertebral and internal carotid artery dissection. With the exception of one patient, the headache and neck pain were unilateral. All patients with vertebral artery dissection complained of posterior cervical or occipital pain. In the cases of internal carotid artery dissection, one patient complained of temporal pain, and one patient with co-existing vertebral artery dissection complained of posterior cervical pain. The mode of onset was acute in five patients, thunderclap in one, and gradual and progressive in one. The pain was severe in all cases. Five patients complained of continuous pain, while two had intermittent pain. The quality of the pain was described as throbbing by five patients and constrictive by two. The headache and neck pain persisted for 1 week or longer in six of the seven patients.

**Conclusion:**

Cervicocephalic arterial dissection should be suspected when patients complain of intense unilateral posterior cervical and occipital pain or temporal pain.

## Introduction

Spontaneous cervicocephalic arterial dissection is an important cause of stroke. The Strategies Against Stroke Study for Young Adults in Japan (SASSY Japan), a multicenter study of the diagnosis, treatment, and prevention strategies for stroke in young adults in Japan, found that 3–4% of strokes occur in people under 50 years of age [1]. Cervicocephalic arterial dissection can cause both ischemic stroke and hemorrhagic stroke. However, spontaneous cervicocephalic arterial dissection presenting only with headache and neck pain has rarely been reported [2–4]. In this study, the clinical features of spontaneous cervicocephalic arterial dissection presenting only with headache and neck pain were investigated.

## Subjects and methods

Between April 2007 and March 2010, 61 patients with spontaneous cervicocephalic arterial dissection (vertebral artery in 41 cases, internal carotid artery in 10 cases, middle cerebral artery in 4 cases, anterior cerebral artery in 3 cases, basilar artery in 3 cases, and posterior cerebral artery in 1 case) were admitted to our stroke center. Of these 61 patients, the clinical presentations were cerebral ischemia in 35 cases, subarachnoid hemorrhage in 13 cases, headache and neck pain only in 7 cases, and asymptomatic in 6 cases. In the asymptomatic cases, cervicocephalic arterial dissection was suspected in the medical checkup of the brain and they were admitted for diagnostic examination. The subjects of this study were the seven cases with headache and neck pain only.

For risk factors, the presence or absence of hypertension, diabetes mellitus, hypercholesterolemia, smoking, and migraine was examined. Diabetes mellitus was defined as blood sugar elevation (either fasting ≥126 mg/dL, 75 g OGTT ≥200 mg/dL, or random ≥200 mg/dL) and HbA1c (international standard) ≥6.5%; hypertension was defined as taking an antihypertensive agent or having blood pressure ≥140/90 mmHg; and hypercholesterolemia was defined as taking a hypolipidemic agent or having total cholesterol ≥220 mg/dL or LDL cholesterol ≥140 mg/dL.

Headache and neck pain were compared for location and mode of onset (thunderclap, acute, gradual, and progressive), severity of pain (severe, moderate), continuousness (intermittent, continuous), and quality (constrictive, throbbing). The mode of onset was classified as thunderclap (sudden onset, intensity of pain peaking at less than 1 min, persisting for 1 h or more), acute (peaking at longer than 1 min but less than 24 h), and progressive (peaking at 24 h or longer). The severity of pain was classified as severe if it was uncontrollable and the patient took analgesics, or moderate if the pain was controllable.

For diagnostic imaging of the cervicocephalic arterial dissection, magnetic resonance imaging/magnetic resonance angiography (MRI/MRA), 3-dimensional computed tomography angiography (3D-CTA), cerebral angiography, and carotid artery ultrasonography were performed, and the diagnostic criteria of SASSY Japan, shown in Table 1, were applied. In all patients, it was confirmed that no trauma (head injury, neck injury, severe cervical distortion) had occurred, and that there was no hereditary abnormality of the arterial wall, such as Marfan’s syndrome or fibromuscular dysplasia. These cases were then diagnosed as spontaneous.

**Table 1:** Diagnostic criteria for cerebral artery dissection

Definite case	
Satisfying any of diagnostic criteria I, II, or III below	
I.	Either intimal flap or double lumen visible on cerebral angiogram
II.	Either intimal flap or double lumen visible on MRI or MRA (tomogram image). Handled identically if the transverse image on 3D-CTA and ultrasound examination is sufficiently delineated, and a clear intimal flap and double lumen are visible
III.	If any of findings IV, V, or VI are seen, and a clear change is seen in the findings over time with repeated imaging examinations. Limited to cases in which a cause other than dissection can be ruled out
Suspected case	
Satisfying any of diagnostic criteria IV, V, or VI below	
IV.	Non-specific findings suggesting arterial dissection (pearl sign, tapered occlusion) are visible on cerebral angiogram other than the findings in I above
V.	Findings are visible on MRA angiogram that appear to correspond to the pearl and string sign, string sign, or tapered occlusion on cerebral angiogram
VI.	Intense signal suggesting intramural hematoma visible on MRI T1-weighted image

The patients were followed after discharge for 1–23 months (mean, 12 months).

## Results

The clinical features of the seven patients (3 males, 4 females) with spontaneous cervicocephalic arterial dissection that presented only as headache and neck pain are shown in Table 2. The patients’ ages ranged from 35 to 79 (mean 51.0 ± 16.2) years (3 patients in their 30s, 2 patients in their 50s, and 1 each in their 60s and 70s). Of the risk factors, five patients had hypercholesterolemia, four had hypertension, three were smokers, and two had migraine.

**Table 2:** Clinical features of seven patients with spontaneous cervicocephalic arterial dissection

Case	Age (years)	Sex	Risk factors	Dissected vessel	Imaging findings	Characteristics of head and neck pain					
						Site	Mode of onset	Severity	Persistence	Nature	Duration
1	35	F	None	Left VA (V4) (intracranial)	MRA, MRA source image: pearl and string sign 3D-CTA: not performed Cerebral angiography: pearl sign Cervical artery ultrasound: not performed	Bilateral posterior cervical, deep orbital	Acute	Severe	Intermittent	Constrictive	9 days
2	51	F	HL	Right VA (V4) (intracranial)	MRA, MRA source image: double lumen 3D-CTA: pearl sign Cerebral angiography: not performed Cervical artery ultrasound: no findings	Right posterior cervical	Acute	Severe	Continuous	Throbbing	25 days
3	79	M	HT, HC, smoker	Right ICA (intracranial)	MRA, MRA source image: pearl and string sign 3D-CTA: not performed Cerebral angiography: not performed Cervical artery ultrasound: no findings	Right temporal	Thunderclap	Severe	Continuous	Constrictive	1 day
4	37	M	HT, smoker	Right VA (V4) (intracranial)	MRA, MRA source image: double lumen 3D-CTA: string sign Cerebral angiography: not performed Cervical artery ultrasound: no finding	Right posterior cervical to occipital	Gradual	Severe	Intermittent	Throbbing	3 months
5	62	M	HT, HC, smoker	Right VA (V4) (intracranial)	MRA, MRA source image: pearl and string sign 3D-CTA: pearl and string sign Cerebral angiography: pearl sign Cervical artery ultrasound: no findings	Right occipital	Acute	Severe	Continuous	Throbbing	14 days
6	36	F	HL, migraine	Left ICA (extracranial) Left VA (V1) (extracranial)	MRA, MRA source image: double lumen 3D-CTA: string sign, intimal flap Cerebral angiography: not performed Cervical artery ultrasound: intimal flap	Left posterior cervical	Acute	Severe	Continuous	Throbbing	20 days
7	56	F	HT, HC, migraine	Right VA (V4) (intracranial)	MRA, MRA source image: string sign 3D-CTA: not performed Cerebral angiography: pearl sign Cervical artery ultrasound: not performed	Right posterior cervical	Acute	Severe	Continuous	Throbbing	3 months

*M* male, *F* female, *HT* hypertension, *HC* hypercholesterolemia, *VA* vertebral artery, *V1, V4* location of vertebral dissection, *ICA* internal carotid artery, *MRA* magnetic resonance angiography, *3D-CTA* 3-dimensional computed tomography angiography

**Fig. 1:**
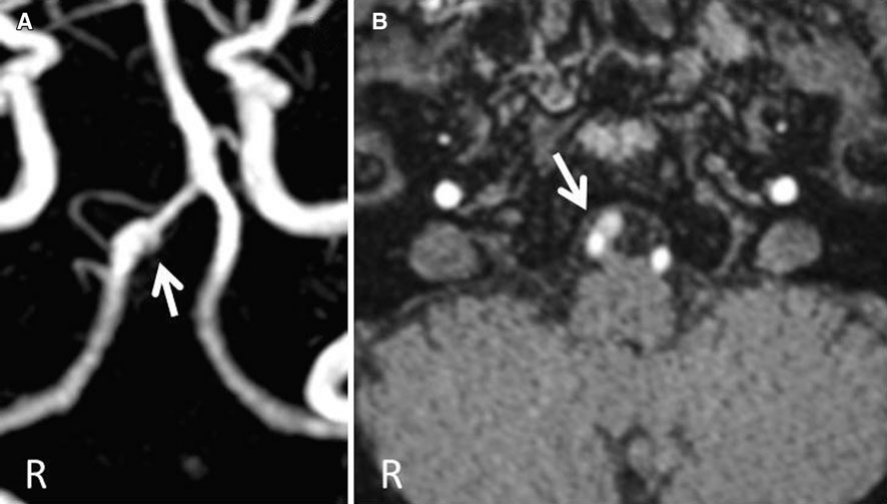
MRA and MRA source image findings in vertebral artery dissection (case 5). **a** Fusiform dilatation is seen at the terminal segment of the right vertebral artery on MRA. **b** A double lumen is seen in the right vertebral artery on the MRA source image

**Fig. 2:**
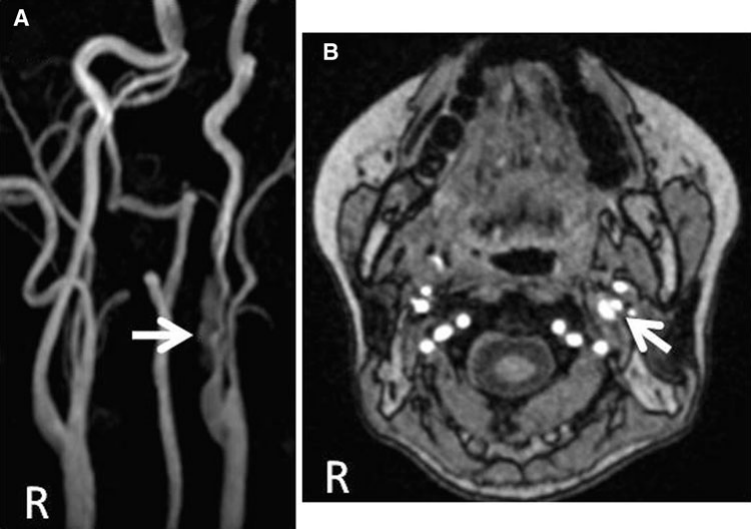
MRA and MRA source image findings in internal carotid artery dissection (case 6). **a** The string sign is visible in the left cervical internal carotid artery on MRA. **b** A double lumen is seen in the left cervical internal carotid artery on the MRA source image

Six patients had vertebral artery dissection, one had internal carotid artery dissection, and one had an association of vertebral and internal carotid artery dissection. Vertebral artery dissection occurred intracranially in five of the six patients and extracranially in the other patient, and internal carotid artery dissection occurred intracranially in one patient and extracranially in one. Typical imaging findings of the vertebral artery dissection and internal carotid artery dissection are shown in Fig. 1 (case 5) and in Fig. 2 (case 6), respectively. Headache and neck pain were localized in all patients, and all patients with vertebral artery dissection complained of posterior cervical and occipital pain. One patient with vertebral artery dissection also complained of deep orbital pain. With regard to laterality, five of six patients complained of pain ipsilateral to the dissected vessel, while one patient complained of bilateral pain. In the cases of internal carotid artery dissection, one patient complained of temporal pain, and one patient with co-existing vertebral artery dissection complained of posterior cervical pain. With regard to the mode of onset, five patients had an acute onset, one patient had thunderclap onset, and one patient had a gradual and progressive onset. The pain was severe in all cases, and because none of the patients could withstand the pain, they presented as emergency patients or night-time outpatients, and all had been taking analgesics. The pain was continuous in five patients and intermittent in two patients. The quality of the pain was reported as throbbing in five patients and constrictive in two patients.

None of the patients experienced stroke (cerebral ischemia or subarachnoid hemorrhage) during hospitalization and follow-up after discharge. With regard to the duration of the cervicocephalic pain, in one patient the condition improved in 1 day, while in the other six patients the pain persisted for 1 week or longer, with a maximum of approximately 3 months. Moreover, improvement vascular dissection such as amelioration of stenosis was seen during the follow-up period in four patients, and all of these patients improved within approximately 3 months.

## Discussion

The seven patients with spontaneous cervicocephalic arterial dissection that presented only with headache and neck pain included six patients with vertebral artery dissection and two patients with internal carotid artery dissection, one of whom had co-existing vertebral artery dissection. Recent clinical studies reported that the nature of spontaneous cervicocephalic arterial dissection in Japan may be very different from that in Europe and North America, since a much higher incidence of vertebral artery dissection cases has been reported in Japanese patients [5, 6]. However, we have already reported [7] that spontaneous cervical internal carotid arterial dissection is not rare in Japan. According to the reports by Arnold et al. [2, 3] and Silbert et al. [4], 10.8% of vertebral artery dissection cases and 2.2% of internal carotid artery dissection cases presented only with headache and neck pain. In the present study, the frequency of patients presenting only with headache and neck pain was 14.6% (6/41) of vertebral artery dissection cases and 20.0% (2/10) of internal carotid artery dissection cases. The frequency in both vessels was greater in the present study, especially in internal carotid artery dissection. One possible reason is that, in the present patients, cervicocephalic arterial dissection was suspected and then diagnosed vigorously by a combination of noninvasive imaging techniques, including MRA, 3D-CTA, and carotid artery ultrasonography.

Headache and neck pain caused by cervicocephalic arterial dissection are thought to be referred pain associated with the nerves distributed to the vessels. The locations of headache and neck pain differ depending on the dissected vessel, and with dissections in the vertebral-basilar arterial system, unilateral cervical pain and occipital pain are frequent, whereas with dissection of the internal carotid artery, temporal pain and facial pain are said to be more frequent than cervical pain [8]. Fay reported that stimulation of carotid artery branching sites caused frontal pain, orbital and periorbital pain, and facial pain [9]. Nichols et al. [10, 11] reported that balloon angioplasty of distal regions of the internal carotid artery and middle cerebral artery caused localized ipsilateral temporal, orbital, and frontal pain, and balloon angioplasty of the vertebral basilar artery caused localized ipsilateral occipital and external cervical posterior pain. In the present patients as well, posterior cervical pain and occipital pain were reported in cases of vertebral artery dissection, and temporal pain was reported in cases of internal carotid artery dissection.

Mokri [8] reported that headache and neck pain caused by cervicocephalic arterial dissection often appear as a sudden unilateral headache and neck pain that the patient has never before experienced. Meanwhile, Arnold et al. [2] stated that no specific headache and neck pain were found in spontaneous cervicocephalic arterial dissection presenting only with head and neck pain. In the present patients, the pain frequently appeared suddenly and was so intense that all of the patients had to take analgesics. In addition, in most cases, the headache and neck pain persisted for 1 week or longer.

The limitation of our study includes patients selection of cervicocephalic arterial dissection was biased because our institution mainly deals with emergency patients with severe symptoms.

Headache and neck pain requiring analgesics are often encountered in daily clinical practice, and identifying spontaneous cervicocephalic arterial dissection in such patients is often very challenging. However, cervicocephalic arterial dissection must be suspected in patients with severe unilateral posterior cervical and occipital pain or temporal pain. In such cases, the combination of multiple diagnostic methods such as MRI/MRA, 3D-CTA, cerebral angiography, carotid artery ultrasonography, and others is needed to make a diagnosis.
